# Supplementary material for: Development of a dual antigen lateral flow immunoassay for detecting Yersinia pestis
Source: PLoS Negl Trop Dis. 2022 Mar 23;16(3):e0010287. doi: 10.1371/journal.pntd.0010287 (PMC8979426; doi:10.1371/journal.pntd.0010287)
Supplement: S1 Fig — Top four LcrV LFI prototypes tested with (A) PBS or (B) Y. pestis Harbin-35 lysate for the detection of native antigen. Test lines were sprayed at 1 mg/mL and 5 uL of gold conjugated mAb (OD540 = 10) was applied. (PDF) [file pntd.0010287.s001.pdf]

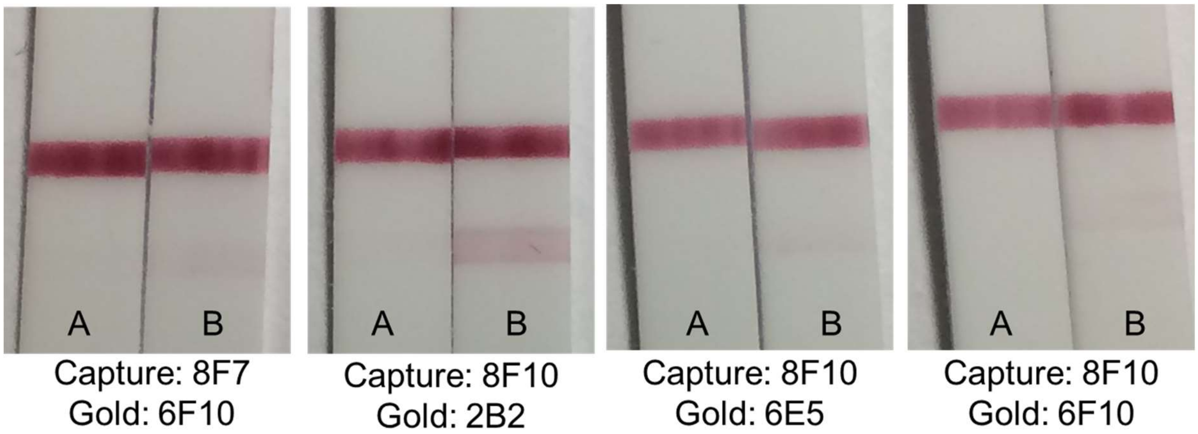

S1 Fig. Top four LcrV LFI prototypes tested with **(A)** PBS or **(B)** *Y. pestis* Harbin-35 lysate for the detection of native antigen. Test lines were sprayed at 1 mg/mL and 5 uL of gold conjugated mAb (OD_{540} = 10) was applied.
