# Supplementary material for: Development of a dual antigen lateral flow immunoassay for detecting Yersinia pestis
Source: PLoS Negl Trop Dis. 2022 Mar 23;16(3):e0010287. doi: 10.1371/journal.pntd.0010287 (PMC8979426; doi:10.1371/journal.pntd.0010287)
Supplement: S2 Fig — Each panel represent a different lot of pool serum from (A-C) Bioreclamation IVT or (D-F) Innovative Resources. Lot numbers are provided for each panel. Assay signal was evaluated and quantitated by optical density using a Qiagen ESE reader. Intensity ≥ 20 mm*mV scores as positive. (PDF) [file pntd.0010287.s002.pdf]

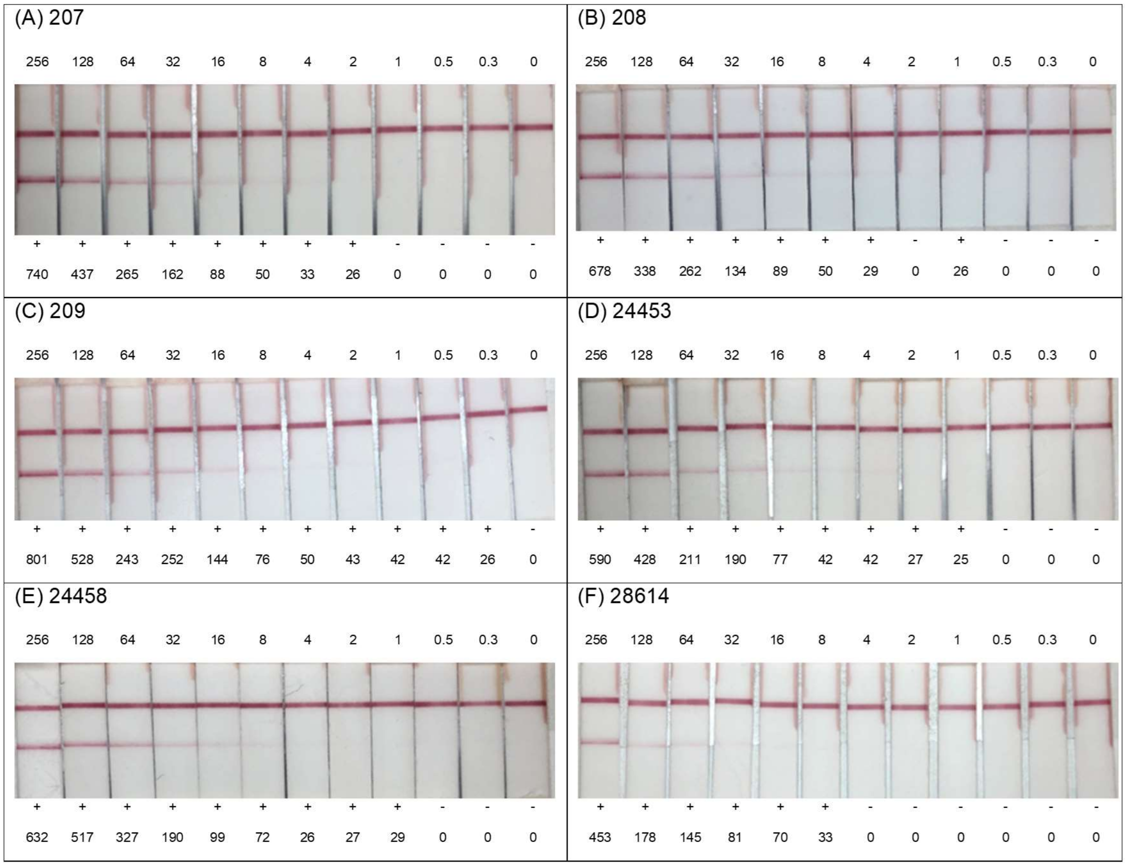

S2 Fig. LFI prototypes (8F10-capture/6F10-detection) were tested with recombinant LcrV in six pools of normal human serum. Each panel represent a different lot of pool serum from **(A-C)** Bioreclamation IVT or **(D-F)** Innovative Resources. Lot numbers are provided for each panel. Assay signal was evaluated and quantitated by optical density using a Qiagen ESE reader. Intensity ≥ 20 mm*mV scores as positive.
