# Supplementary material for: Development of a dual antigen lateral flow immunoassay for detecting Yersinia pestis
Source: PLoS Negl Trop Dis. 2022 Mar 23;16(3):e0010287. doi: 10.1371/journal.pntd.0010287 (PMC8979426; doi:10.1371/journal.pntd.0010287)
Supplement: S3 Fig — Each panel represent a different lot of pool serum from (A-C) Bioreclamation IVT or (D-F) Innovative Resources. Lot numbers are provided for each panel. Assay signal was evaluated and quantitated by optical density using a Qiagen ESE reader. Intensity ≥ 20 mm*mV scores as positive. (PDF) [file pntd.0010287.s003.pdf]

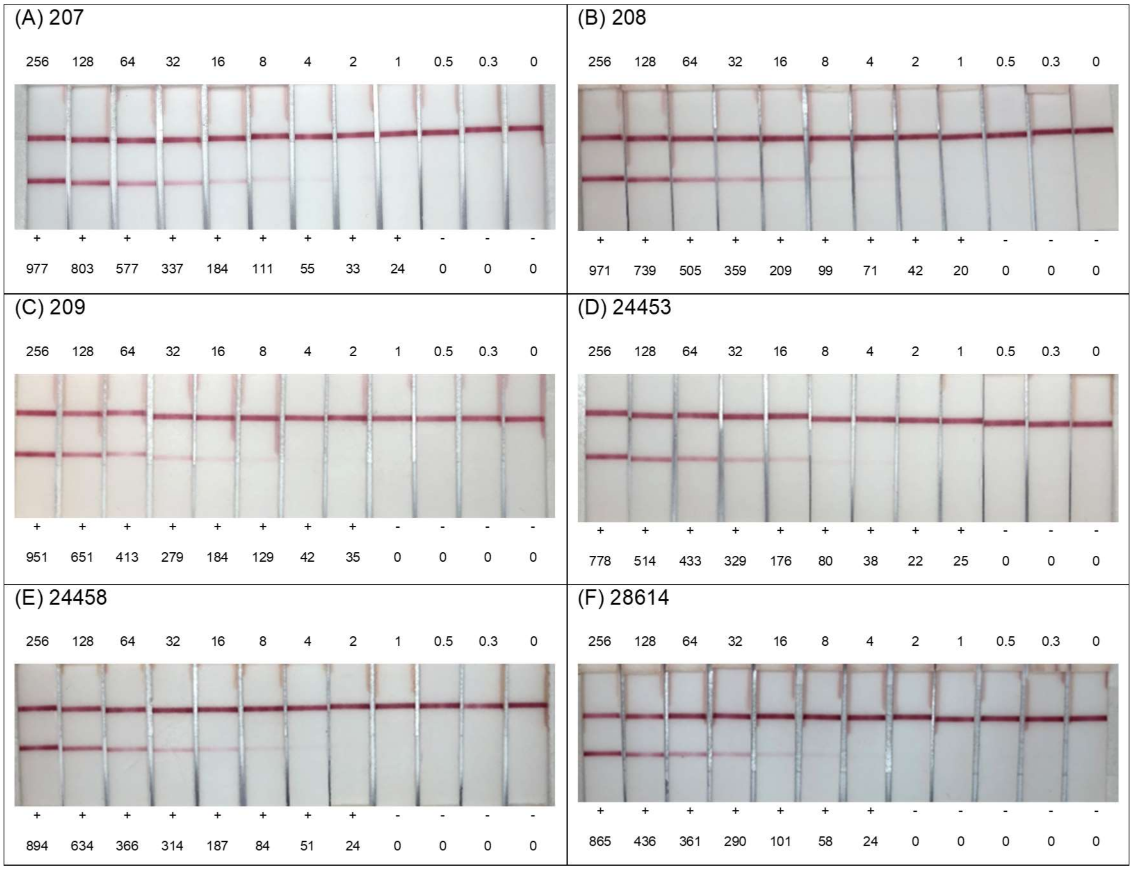

S3 Fig. F1 prototypes (11C7-capture/3F2-detection) were tested with recombinant F1 in six pools of normal human serum. Each panel represent a different lot of pool serum from **(A-C)** Bioreclamation IVT or **(D-F)** Innovative Resources. Lot numbers are provided for each panel. Assay signal was evaluated and quantitated by optical density using a Qiagen ESE reader. Intensity ≥ 20 mm*mV scores as positive.
