# Supplementary material for: Development of a dual antigen lateral flow immunoassay for detecting Yersinia pestis
Source: PLoS Negl Trop Dis. 2022 Mar 23;16(3):e0010287. doi: 10.1371/journal.pntd.0010287 (PMC8979426; doi:10.1371/journal.pntd.0010287)
Supplement: S4 Fig — Values shown are the concentrations of (A) recombinant LcrV and (B) recombinant F1 in ng/ml at five times background for each mAb pair. The values represent the mean of two independent ELISAs (each performed in biological triplicates). (PDF) [file pntd.0010287.s004.pdf]

(A)

	Detection (HRP conjugated) mAb								
Capture mAb		2B2	4E8	5D3	6E5	6F10	8F3	8F7	8F10
	**2B2**	4.2	10	27	5.9	5.8	9.3	ND	40
	**4E8**	10	16	48	23	17	2.2	2.5	1.8
	**5D3**	13	23	110	24	19	3.5	2.5	1.9
	**6E5**	24	28	37	34	22	3.1	0.8	0.6
	**6F10**	7.6	16	40	11	11	2.1	0.8	0.6
	**8F3**	0.8	2.1	3.9	1.1	0.9	20	12	6.3
	**8F7**	0.8	2.3	9.6	32	52	130	32	7.7
	**8F10**	1.1	2.7	5.6	4.7	2.3	51	12	14

(B)

	Detection (HRP conjugated) mAb								
Capture mAb		3F2	4E5	4F12	5E10	10D9	11B8	11C7	15C4
	**3F2**	2.9	1.1	6.0	1.3	2.8	1.5	0.7	1.1
	**4E5**	2.8	2.3	2.5	7.9	1.9	16	2.8	1.9
	**4F12**	10	1.5	5.5	2.2	1.9	3.4	0.8	1.6
	**5E10**	7.8	5.0	3.1	2.9	3.4	4.7	2.7	2.6
	**10D9**	2.5	1.2	2.2	3.2	4.8	1.7	0.6	1.3
	**11B8**	2.4	4.0	2.1	3.0	3.2	1.6	0.6	2.1
	**11C7**	15	2.3	2.7	6.0	2.8	1.6	1.5	1.9
	**15C4**	4.3	4.9	3.1	5.5	2.6	2.5	0.9	3.6

S4 Fig. Preliminary screen to identify top performing antigen-capture ELISA mAb pairs. Values shown are the concentrations of **(A)** recombinant LcrV and **(B)** recombinant F1 in ng/ml at five times background for each mAb pair. The values represent the mean of two independent ELISAs (each performed in biological triplicates).
