# Supplementary material for: Development of a dual antigen lateral flow immunoassay for detecting Yersinia pestis
Source: PLoS Negl Trop Dis. 2022 Mar 23;16(3):e0010287. doi: 10.1371/journal.pntd.0010287 (PMC8979426; doi:10.1371/journal.pntd.0010287)
Supplement: S5 Fig — Antigen-capture ELISAs were performed to determine the limits of detection (LOD) for recombinant (A & B) LcrV and (C & D) F1. LODs were calculated using the linear regression of the optimized ELISA conditions to determine the concentration of recombinant protein in ng/ml at two-fold background. The values represent means of three independent ELISAs (each performed in biological triplicates). (PDF) [file pntd.0010287.s005.pdf]

(A)

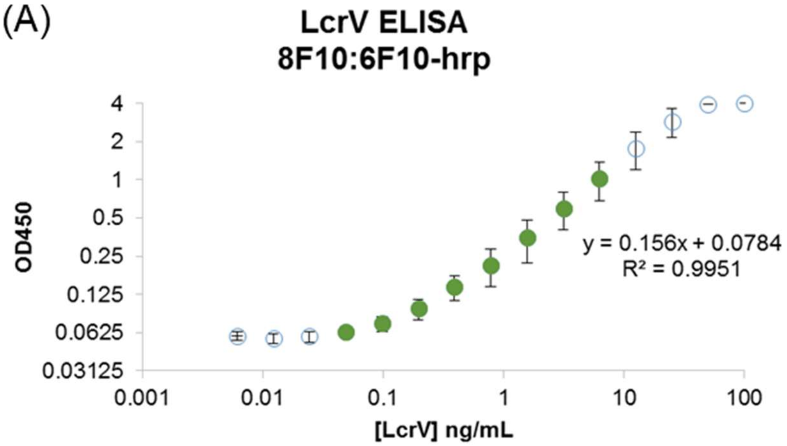

LcrV ELISA
8F10:6F10-hrp
4
2
1
0.5
0.25
0.125
0.0625
0.03125
OD450
0.001
0.01
0.1
1
10
100
[LcrV] ng/mL
y = 0.156x + 0.0784
R² = 0.9951

(B)

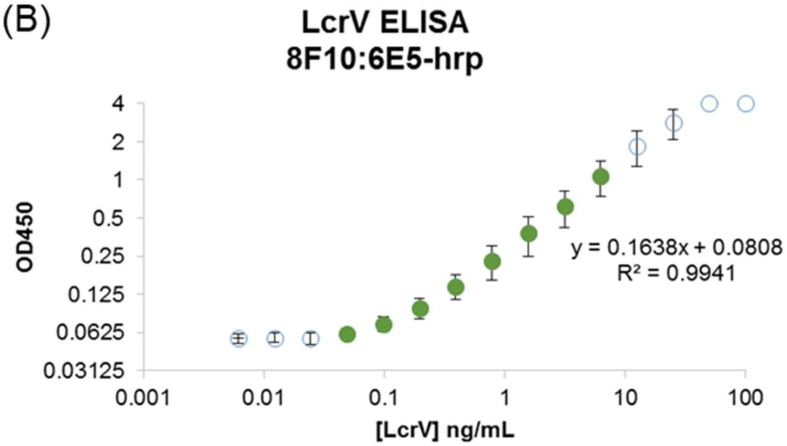

LcrV ELISA
8F10:6E5-hrp
4
2
1
0.5
0.25
0.125
0.0625
0.03125
OD450
0.001
0.01
0.1
1
10
100
[LcrV] ng/mL
y = 0.1638x + 0.0808
R² = 0.9941

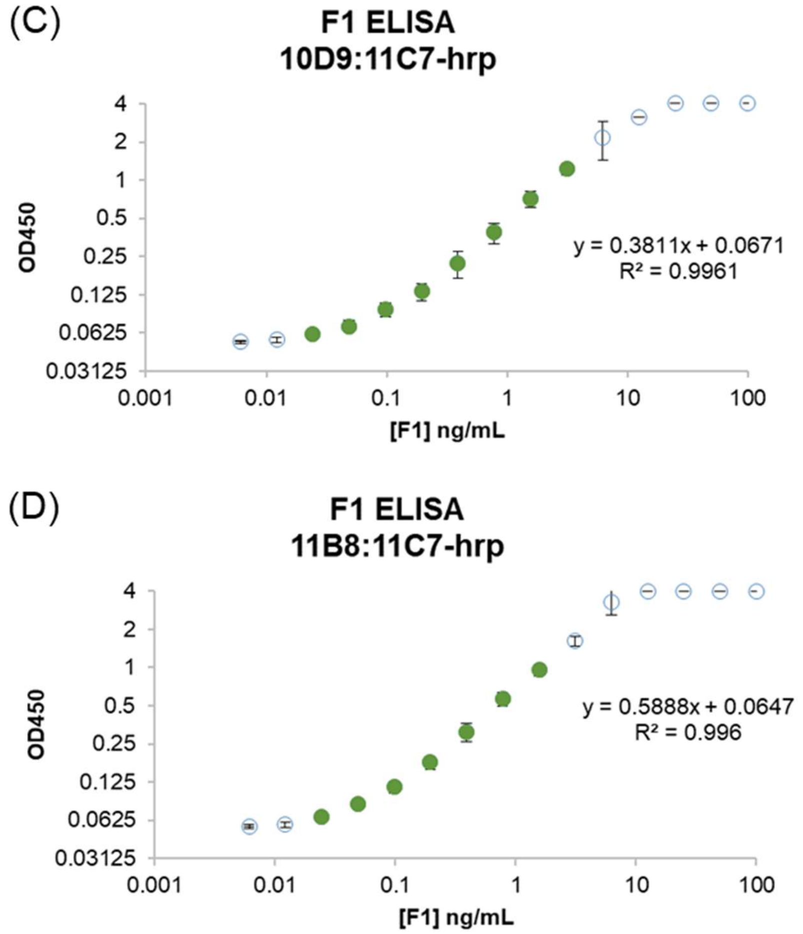

S5 Fig. Antigen-capture ELISAs were performed to determine the limits of detection (LOD) for recombinant **(A)** LcrV and **(B)** F1. Values were calculated as the concentration of recombinant protein in ng/ml at five times background. The values represent means of two independent ELISAs (each performed in biological triplicates).
