# Supplementary material for: Development of a dual antigen lateral flow immunoassay for detecting Yersinia pestis
Source: PLoS Negl Trop Dis. 2022 Mar 23;16(3):e0010287. doi: 10.1371/journal.pntd.0010287 (PMC8979426; doi:10.1371/journal.pntd.0010287)
Supplement: S1 Table — (PDF) [file pntd.0010287.s006.pdf]

S1 Table. Primers for cloning LcrV and F1 genes from *Y. pestis* Harbin-35 into the pQe-30 Xa vector.

Gene		***Primer***	***Tm (°C)***
lcrv	F	5'-GGTATCGAGGGAAGGATGATTAGAGCCTACGAACA-3'	66
	R	5'-GTCCAAGCTCAGCTATCATTTACCAGACGTGTCAT-3'	64
caf1	F	5'-GGTATCGAGGGAAGGGCAGATTTAACTGCAAGCAC-3'	67
	R	5'-GTCCAAGCTCAGCTATTATTGGTTAGATACGGTTAC-3'	63
