# Supplementary material for: Development of a dual antigen lateral flow immunoassay for detecting Yersinia pestis
Source: PLoS Negl Trop Dis. 2022 Mar 23;16(3):e0010287. doi: 10.1371/journal.pntd.0010287 (PMC8979426; doi:10.1371/journal.pntd.0010287)
Supplement: S2 Table — (PDF) [file pntd.0010287.s007.pdf]

S2 Table. Summary of lateral flow immunoassay components evaluated

Conjugate Release Pads	Nitrocellulose Membranes	Wicking Pads	Surfactants	Buffers
Ahlstrom Munksjo 6613H	UniSart CN140	C083 Cellulose Fiber	10G	Borate Buffer
Ahlstrom Munksjo 8951	UniSart CN95	Whatman CF4	F127	Carbonate-Bicarbonate Buffer
Ahlstrom Munksjo 8964	Whatman FF120HP	Whatman CF5	PVP40	Phosphate buffered Saline
Whatman Fusion 5	Whatman FF170HP	Whatman CF6	Tergitol	Tris buffered Saline
	Whatman FF80HP	Whatman CF7	Triton-X-100	
			Tween-20	
