# Supplementary material for: Development of a dual antigen lateral flow immunoassay for detecting Yersinia pestis
Source: PLoS Negl Trop Dis. 2022 Mar 23;16(3):e0010287. doi: 10.1371/journal.pntd.0010287 (PMC8979426; doi:10.1371/journal.pntd.0010287)
Supplement: S3 Table — (PDF) [file pntd.0010287.s008.pdf]

S3 Table. Assay signal intensity of top mAb pairs by LFI for LcrV at 100 ng/mL

Capture mAb	Detection mAb	100 ng/mL LcrV	Chase only	Difference
6E5	8F10	500	277	223
4E8	8F10	452	238	214
2B2	8F10	488	278	210
8F7	6E5	315	131	184
8F10	6E5	253	94	159
8F10	6F10	297	164	133
8F10	2B2	234	106	128
8F7	2B2	244	137	107
8F7	4E8	330	223	107
8F7	6F10	260	178	82
