# Supplementary material for: Development of a dual antigen lateral flow immunoassay for detecting Yersinia pestis
Source: PLoS Negl Trop Dis. 2022 Mar 23;16(3):e0010287. doi: 10.1371/journal.pntd.0010287 (PMC8979426; doi:10.1371/journal.pntd.0010287)
Supplement: S4 Table — (PDF) [file pntd.0010287.s009.pdf]

S4 Table. Assay signal intensity of top mAb pairs by LFI for F1 at 100 ng/mL

Capture mAb	Detection mAb	100 ng/mL F1	Chase only	Difference
11C7	4E5	834	71	763
5E10	11C7	725	0	725
10D9	3F2	709	0	709
11C7	5E10	665	0	665
3F2	4E5	616	0	616
11C7	15C4	599	0	599
11C7	11B8	599	0	599
11C7	11C7	591	0	591
11C7	3F2	587	0	587
4E5	3F2	540	0	540
11C7	4F12	495	0	495
15C4	3F2	539	53	487
11B8	4E5	508	64	444
9B7	3F2	439	0	439
5E10	3F2	434	0	434
11C7	10D9	434	0	434
11C7	9B7	427	0	427
3F2	15C4	423	0	423
5E10	11B8	542	124	418
11B8	10D9	416	0	416
