# Supplementary material for: Development of a dual antigen lateral flow immunoassay for detecting Yersinia pestis
Source: PLoS Negl Trop Dis. 2022 Mar 23;16(3):e0010287. doi: 10.1371/journal.pntd.0010287 (PMC8979426; doi:10.1371/journal.pntd.0010287)
Supplement: S5 Table — (PDF) [file pntd.0010287.s010.pdf]

S5 Table. Preliminary assay reactivity of top mAb pairs by LFI for F1 at 1 ng/mL

Capture mAb	Detection mAb	1 ng/mL F1	Chase only	Difference
11C7	4E5	500	112	388
11C7	5E10	149	33	116
11C7	3F2	115	0	115
11C7	15C4	93	0	93
4E5	3F2	89	0	89
11C7	11B8	138	53	86
10D9	3F2	57	0	57
5E10	11C7	118	106	13
3F2	4E5	125	139	-14
