# Supplementary material for: Development of a dual antigen lateral flow immunoassay for detecting Yersinia pestis
Source: PLoS Negl Trop Dis. 2022 Mar 23;16(3):e0010287. doi: 10.1371/journal.pntd.0010287 (PMC8979426; doi:10.1371/journal.pntd.0010287)
Supplement: Attachment — Submitted filename: Comments.docx [file pntd.0010287.s011.docx]

**General comments**

The article presented here describes the development of monoclonal antibodies and immunological tests (LFI and ELISA) for the detection of two *Yersinia pestis* antigens (LcrV and F1). The article is interesting but would deserve to be completed by additional data.

The main criticism I can make is that the authors performed the selection of their antibodies on recombinant proteins or bacterial lysates heated at 80°C for 2h, which means that the antibodies were not properly evaluated during their selection against not heat-denatured native proteins. The authors should perform the antibody selection experiments using the bacterial culture supernatants, which contain both LcrV and F1, and have the advantage of not being thermally denatured (filtration allows them to be used without thermal inactivation). The authors may then be able to confirm (or not) that the selected antibodies are indeed the best against the two natural proteins in their native conformation.

On the other hand, the authors do not provide any details on the determination of the LoD of the ELISA tests, which seem to have come out of nowhere. It is also vaguely mentioned that the sensitivity of the tests is better than a commercial ELISA, but it is not known which commercial test, targeting which protein, and how the evaluation was performed. The authors should show comparative experiments.

**In details :**

Line 67 : correct by « classify « ?

Line 169 : change by « Animals were used in this study to produce… »

Cells are inactivated at 80°C : only non conformational antibodies are selected

Lines 222 and 223 replace (BSL2) by (BSL3) (or I misunderstood the sentence)

Screening of the mAbs : why don’t the authors select the best mAbs also using native LcrV coming from Y. pestis superantants ?

Lines 335, 338, 396 and all along the text : it should be precised that the native protein is also unfolded (heating at 80°C for 2 hours) and thus does not reflect the « true » native folded proteins. Please find another word to replace native such as « natural » ?

Line 337 : samples have been heated (80°C for 2 hours) before WB which is highly denaturant (10% SDS), thus the multimeric forms that are detected are most probably not the native ones found in the culture but reflect more agregative states of the protein. Moreover the signals obtained are relatively weak for aggregates compared to those for monomeric LcrV. Those aggregates do not disappear in non reduced gel, in agreement with the fact that they are probably artificial aggregates made by heating. Please modify the sentence.

Line 343 : reducing conditions do not disrupt protein-protein interactions, they only « disrupt » disulfides bonds. There are numerous protein-protein interactions that do not involve disulfide bonds. Please correct.

mAbs were produced against soluble recombinant proteins produced in the cytosol of E. coli. Those proteins are not supposed to have disulfides bonds (except if they have been refolded in the conditions allowing the formation of disulfide bonds, which is not mentioned in the protocol of production of recombinant proteins in the experimental procedures). How do you explain the results obtained in non reduced or reduced conditions, with mAbs that are not supposed to be influenced by the disulfide bonds ?

WB against F1 protein in reducing conditions : please show the entire WB as it is done in non reducing conditions.

Line 348. Please explain. Would it be that those antibodies have their epitopes straddling both monomers ?

Western blots and LFI : authors should have tested their antibodies with bacterial supernatants, and not only bacterial lysates for the selection of best mAbs pairs. Recognition of heated natural or recombinant protein could be different from the recognition of the native correctly refolded protein

Table 2 : correct K_D_

Line 432 : correct µg/mL instead of mg/mL

ELISA sensitivity : please show a graph with the curves obtained (and which fit has been used), and the limit of detection. Please explain in the text how was determined the LoD. If the LoD is a theoretical LoD, please precise it (indicate theoretical LoD in the text). Give also in the text the way of calculating the LoD (NS mean + X SD of the NS). Please indicate the number of NS values that have been taken into account for the calculation of LoD. If LoD is an experimental LoD, please detail the way the tests have been performed and how was determined the experimental LoD

Line 439 : please indicate the brand and name of the commercial assay and which antigen is targeted (LcrV or F1 ?) and show the experimental data for the comparison of this assay with the in house assay described in the article. If no experimental data are available (in the same experiment, with the same proteins), the sentence should be suppressed.

If experimental data are available (and shown), it should be also precised if the LoD of commercial assay has been evaluated with recombinant proteins or with natural non denatured protein)

Add a legend to table 4 explaining how the LoDs have been calculated

Line 451 : avoid repetition present/presentations. Replace presentations by symptoms ?

Line 454 : please justify « growing » and give a reference for that.

Line 458 : suppress « expanded » : only three isolates have been tested

Lines 477-478 : authors can not conclude, based on the results on three strains that the mAb reactivity is against most if not all strains. Please modify the sentence (could be the replacement of « is » by « might be »), and suppress « However » in the following sentence.

Line 511 : 48h post inhalation is late. Please add a sentence to explain that the challenge would be to detect antigens very early (in the first 24h post infection) in order to give the appropriate treatment asap.

Line 547 or line 593 : add something about the non surprising cross-reactivity with Y. pseudotubersulosis and how it could be managed on field.

Line 562 : leaky expression would very probably be not sufficient for detection by LFI

Line 574 : correct « pentameric » and not « pentametric »

Line 577 : see the comment above and please suppress this sentence

Line 579 : please correct « result » instead of « results »

Line 587 : please give the references and the LoD of the commercially available assays.. Authors should know that the experiments are the only way to prove it : antigens should be the same for an exact comparison.

Line 18 supplementary : correct « represents »

Supp figure 1 : please add also the tests realized for the selection of anti F1 antibodies. In particular, add the pair that has been selected further (11C7/3F2)
