# Supplementary material for: Development of a dual antigen lateral flow immunoassay for detecting Yersinia pestis
Source: PLoS Negl Trop Dis. 2022 Mar 23;16(3):e0010287. doi: 10.1371/journal.pntd.0010287 (PMC8979426; doi:10.1371/journal.pntd.0010287)
Supplement: Attachment — Submitted filename: 20220124_ResponsestoReviewers.docx [file pntd.0010287.s012.docx]

**Response to academic editor**

Dear Academic Editor,

We would like to thank the reviewers for a thoughtful analysis of the manuscript. We have endeavored to address the comments provided by all the reviewers, and hope that these changes are acceptable.

The comments provided by the reviewers are valid and we believe the results generated thus far in this study merit publication due to their novelty. Current rapid diagnostic tests (RDT) used in the field detect for F1, a highly expressed antigen. Of concern are F1 deficient mutants which can also cause disease. Although F1 has been used as a diagnostic antigen for plague, lesser is known about LcrV as a viable biomarker. Detection of LcrV has been demonstrated in mouse models of infection; however the detection in human cases has not been explored and is warranted. This study is the first instance to our knowledge of multiplexing these two antigens for identification of plague. As described in the manuscript, these assay prototypes have analytical sensitivities within the published relative range and are specific when compared to other Tier 1 bacterial select agents.

At this point our assays are prototypes and would need to undergo much more testing to validate their utility in the field. There were a number of suggestions by reviewers for additional analysis which we hope to accomplish. Due to biosafety regulations, patient samples are challenging to acquire and therefore the best available options to test our assays were with recombinant protein and select agent exempt strains of *Yersinia pestis.* We recognize that this limits our ability to validate the assays for clinical use. As we have shown in the study, our assay prototypes are reactive with *Y. pestis* lysates prepared from clinical isolates; therefore it is likely that future studies will be able to show reactivity with plague patient samples.

We agree that Reviewer 3’s suggestion of testing other clinical matrices (i.e. whole blood, bubo aspirate, sputum) is warranted. Optimization in serum was selected based on previously published data on both antigens of interest. We are attempting organize collaborations to test patient samples and/or animal model samples to further evaluate assay specificity and sensitivity.

The quantitative ELISA for detection of *Y. pestis* LcrV and F1 developed in this study is a valuable tool which could be applied by researchers to further characterize the dynamics of the antigen during plague infection, as well as in clinical and environmental studies.

We feel that publication of these assays would provide researchers with access to a way to easily quantify these biomarkers in their own studies. Furthermore, novel antibodies such as those produced here could be extremely useful to researchers for a variety of applications in plague research. As touched upon in the discussion section of the manuscript, it is possible that the antibodies described may be useful for therapeutic uses.

**Reviewer 1**

**Comment:** Line 67 : correct by « classify « ?

**Response**: These corrections were made.

**Comment:** Line 169 : change by « Animals were used in this study to produce… »

**Response**: These corrections were made.

**Comment:** Lines 222 and 223 replace (BSL2) by (BSL3) (or I misunderstood the sentence)

**Response**: These corrections were made. These experiments were run at BSL2

**Comment:** Screening of the mAbs : why don’t the authors select the best mAbs also using native LcrV coming from Y. pestis superantants ?

**Response**: Due to constraints with biosafety regulation, mAbs were selected using recombinant protein. Antibody pairs were down selected using whole cell lysates prepared at BSL2. Supernatant collected from *Y. pestis* cultures grown in BHI broth at 37°C did not contain enough LcrV to evaluate by standard immunoassay techniques (Western blots and ELISA).

**Comment:** Lines 335, 338, 396 and all along the text : it should be precised that the native protein is also unfolded (heating at 80°C for 2 hours) and thus does not reflect the « true » native folded proteins. Please find another word to replace native such as « natural » ?

**Response:** This has been corrected throughout the text.

**Comment:** Line 337 : samples have been heated (80°C for 2 hours) before WB which is highly denaturant (10% SDS), thus the multimeric forms that are detected are most probably not the native ones found in the culture but reflect more agregative states of the protein. Moreover the signals obtained are relatively weak for aggregates compared to those for monomeric LcrV. Those aggregates do not disappear in non reduced gel, in agreement with the fact that they are probably artificial aggregates made by heating. Please modify the sentence.

**Response**: Supernatant collected from *Y. pestis* cultures grown in BHI broth at 37°C did not contain enough LcrV to evaluate the effects of heat on protein aggregation. The sentence has been modified to omit reference to multimeric structures.

**Comment:** Line 343 : reducing conditions do not disrupt protein-protein interactions, they only « disrupt » disulfides bonds. There are numerous protein-protein interactions that do not involve disulfide bonds. Please correct.

**Response**: Modifications to the text. Samples for non-reducing Western blots were also not treated with heat. The F1 filamentous structure has been found to be stable in the presence of SDS at temperatures <70°C as the protein (Soliakov 2010). Despite the Western blots shown are SDS-PAGE gels, these conditions such a more native structure, yielding the banding pattern in the non-reduced image.

**Comment:** mAbs were produced against soluble recombinant proteins produced in the cytosol of E. coli. Those proteins are not supposed to have disulfides bonds (except if they have been refolded in the conditions allowing the formation of disulfide bonds, which is not mentioned in the protocol of production of recombinant proteins in the experimental procedures). How do you explain the results obtained in non reduced or reduced conditions, with mAbs that are not supposed to be influenced by the disulfide bonds ?

**Response**: see above

**Comment:** WB against F1 protein in reducing conditions : please show the entire WB as it is done in non reducing conditions.

**Response**: This has been corrected.

**Comment:** Line 348. Please explain. Would it be that those antibodies have their epitopes straddling both monomers ?

**Response**: The F1 capsule is formed of polymerized F1 subunits. Based on the banding pattern of 9B7 and 12F5, these mAbs are not reactive to individual F1 subunits or dimerized F1. This may suggest that alternating F1 subunits contribute to tertiary structure of the F1 capsule and the epitopes of these mAbs are at these interfaces. This has been updated for clarity to:

“Reactivity of mAbs 9B7 and 12F5 may suggest binding only occurs to a specific epitope formed in the F1 capsule between at least three or more subunits (>46.5 kDa).”

**Comment:** Western blots and LFI : authors should have tested their antibodies with bacterial supernatants, and not only bacterial lysates for the selection of best mAbs pairs. Recognition of heated natural or recombinant protein could be different from the recognition of the native correctly refolded protein

**Response**: Supernatant collected from *Y. pestis* cultures grown in BHI broth at 37°C did not contain enough LcrV to evaluate the effects of heat on protein aggregation. The sentence has been modified to omit reference to multimeric structures. Optimization steps were done with recombinant protein and validated with heat-inactivated samples to follow biosafety protocols in place.

**Comment:** Table 2 : correct KD

**Response**: This has been corrected.

**Comment:** Line 432 : correct µg/mL instead of mg/mL

**Response**: This has been corrected.

**Comment:** ELISA sensitivity : please show a graph with the curves obtained (and which fit has been used), and the limit of detection. Please explain in the text how was determined the LoD. If the LoD is a theoretical LoD, please precise it (indicate theoretical LoD in the text). Give also in the text the way of calculating the LoD (NS mean + X SD of the NS). Please indicate the number of NS values that have been taken into account for the calculation of LoD. If LoD is an experimental LoD, please detail the way the tests have been performed and how was determined the experimental LoD

**Response**: Graph with curves have been included in supplemental figures. The theoretical limit of detection was defined in the methods and materials as two-fold background signal

**Comment:** Line 439 : please indicate the brand and name of the commercial assay and which antigen is targeted (LcrV or F1 ?) and show the experimental data for the comparison of this assay with the in house assay described in the article. If no experimental data are available (in the same experiment, with the same proteins), the sentence should be suppressed.

**Response**: Sentence has been suppressed as comparative analyses were not run.

**Comment:** If experimental data are available (and shown), it should be also precised if the LoD of commercial assay has been evaluated with recombinant proteins or with natural non denatured protein)

**Response**: Sentence has been removed as comparative analyses were not run.

**Comment:** Add a legend to table 4 explaining how the LoDs have been calculated

**Response**: This has been corrected.

**Comment:** Line 451 : avoid repetition present/presentations. Replace presentations by symptoms ?

**Response**: This has been corrected.

**Comment:** Line 454 : please justify « growing » and give a reference for that.

**Response**: The sentence has been modified to exclude growing.

**Comment:** Line 458 : suppress « expanded » : only three isolates have been tested

**Response**: This has been corrected.

**Comment:** Lines 477-478 : authors can not conclude, based on the results on three strains that the mAb reactivity is against most if not all strains. Please modify the sentence (could be the replacement of « is » by « might be »), and suppress « However » in the following sentence.

**Response**: This has been corrected.

**Comment:** Line 511 : 48h post inhalation is late. Please add a sentence to explain that the challenge would be to detect antigens very early (in the first 24h post infection) in order to give the appropriate treatment asap.

**Response**: Modifications have been made. The text now includes the need to test early infection samples and how the immunoassay described can assist with diagnostic antigen validation studies as a future direction.

**Comment:** Line 547 or line 593 : add something about the non surprising cross-reactivity with Y. pseudotubersulosis and how it could be managed on field.

**Response**: The following text has been added:

It is no surprise that the LcrV prototype was positive for Y. pseudotuberculosis as the homology is 97% (Daniel 2009). In cases where plague is suspected, a positive result for either F1 or LcrV should prompt immediate treatment.

**Comment:** Line 562 : leaky expression would very probably be not sufficient for detection by LFI

**Response**: Sentence has been modified to present the possibility of testing insect vector by LFIs.

**Comment:** Line 574 : correct « pentameric » and not « pentametric »

**Response**: This has been corrected.

**Comment:** Line 577 : see the comment above and please suppress this sentence

**Response**: This has been corrected.

**Comment:** Line 579 : please correct « result » instead of « results »

**Response**: This has been corrected.

**Comment:** Line 587 : please give the references and the LoD of the commercially available assays.. Authors should know that the experiments are the only way to prove it : antigens should be the same for an exact comparison.

**Response**: Modifications to the text were made to acknowledge LoD limitations and to remove the comparative statement. Limitations are acknowledged and future directions to compare assays are presented.

**Comment:** Line 18 supplementary : correct « represents »

**Response**: This has been corrected.

**Comment:** Supp figure 1 : please add also the tests realized for the selection of anti F1 antibodies. In particular, add the pair that has been selected further (11C7/3F2)

**Response**: mAb pairs for the F1 prototype were selected by top performing pairs at 1 ng/mL of recombinant protein. Supplemental table 5 was added to show the preliminary data of the top 8 pairs at a lower concentration of F1 antigen.

**Reviewer 2**

**Comment:** Line 38: Full stop omitted after the Y for Y. pestis

**Response**: This has been corrected.

**Comment:** Line 106: Define F1 minus strain at first use.

**Response**: Text has been modified to define F1 minus strains as being deficient of the capsule

**Comment:** Line 117: Expression of F1 is temperature-induced at 33C and above.

**Response**: Modifications to the temperature and the proper citation have been made.

**Comment:** Line 171: The word female should not be a capital. “Twenty Female CD1 mice..”.

**Response**: This has been modified.

**Comment:** Line 179: The word “or” should be “for”. “to account or variability”

**Response**: This has been corrected.

**Comment:** Line 222: BSL2 should be BSL3. “biosafety level 3 (BSL2)”

**Response**: This has been corrected.

**Comment:** Line 224: “Francisella tularensis” should be abbreviated to “F. tularensis”. First use of full scientific name was in line 218.

**Response**: This has been corrected.

**Comment:** Line 225: “Bacillus anthracis” should be abbreviated to “B. anthracis”. First use of full scientific name was in line 219.

**Response**: This has been corrected.

**Comment:** Line 246: Write out SDS.

**Response**: This has been corrected.

**Comment:** Line 262: “ten” should be in numerals. Numbers from ten and above should be written as numerals.

**Response**: This has been corrected.

**Comment:** Line 266: “40uL”. Don’t start a sentence with numerals.

**Response**: This has been corrected.

**Comment:** Line 294: “2” should be written in words. Numbers from zero to nine should be written in words.

**Response**: This has been corrected.

**Comment:** Line 349: “3” should be written in words. As above.

**Response**: This has been corrected.

**Comment:** Line 603-809: Check reference list. Full names of authors not listed for a number of publications i.e. references 17 (line 639), 20 (line 648), 21 (line 651), 33 (line 685), 47 (line 725), 54 (line 745), 58 (line 756), 59 (line 760), 62 (line 768), 65 (line 775), 66 (line 778), etc.

**Response**: This has been corrected.

**Comment:** Lines 205, 227, 238. 259, 260, 262, 263, 399, 432: There should be a space between the number and the unit of measure e.g. line 205 - 450 nm, line 259 - 1 mg/mL etc.

**Response**: This has been corrected.

**Reviewer 3**

All appropriate changes were made in the text regarding grammatical errors.

In the materials and methods section, additional information on cell culture has been described. The limitations of using serum as the clinical matrix has been addressed. Justification for serum testing based on published literature have been included. The theoretic limit of detection (LoD) of the ELISA has been defined in the M&M. Supplemental figures have been included to show graphs of the optimization ELISA pairs and well as the linear regression in which was used to define the theoretical LoD. Information on the components tested for the lateral flow immunoassays were added in Supplemental table 2. Placement of the F1 and LcrV test lines were justified in the results section.

Additional mAbs were isolated from the F1-V immunizations, however many of these mAbs were not reactive to the individual recombinant subunits (F1 and LcrV). The present study only evaluated antibodies that were reactive to the individual subunits; therefore, 2B2 and 3A2 were confirmed to be reactive to both F1-V and its individual target.

For Figure 3, test line reader intensities were chosen over developers’ visual readings to remove human bias. Values shown are averages between LFI replicates. Of interest would be to determine the minimum reader value in which neophytes in the field can distinguish a positive signal to give developers a baseline.

We acknowledge the preliminary nature of the prototypes describe and are looking into collaborations to validate these assays. Target product profile (TPP) has not been defined as these prototypes are in early development. Further investigation using clinical samples will need to be done to determine the viability of LcrV. Two of each assay type (LFI and ELISA) are described to allow for flexibility in the next stages of development. In the present study, the assays were optimized to analytical sensitivity and specificity. For a commercialized product, additional studies will need to be conducted to establish the TPP. These research tools will be helpful to further elucidate these targets in clinical specimens, and will guide the development and TPP of each assay.
